# Supplementary material for: Cyst-independent oocyte phagocytosis builds the female reproductive reserve in mice
Source: EMBO Rep. 2025 Dec 8;27(1):230–55. doi: 10.1038/s44319-025-00663-7 (PMC12796176; doi:10.1038/s44319-025-00663-7)
Supplement: Supplementary file 1 — Appendix [file 44319_2025_663_MOESM1_ESM.pdf]

Appendix

Table of content

Page 2: **Appendix Figure S1.** Labeling germ cell membrane in the *Oct4-CreER*^{T2}*;mTmG* ovaries.
Page 3: **Appendix Figure S2.** Time-lapse imaging to trace the development of a group of oocytes.
Page 4: **Appendix Figure S3.** Quantification of the developmental dynamics and cellular behaviors of oocytes during ovariogenesis.
Page 5: **Appendix Figure S4.** Recording and analyzing the progress of oocyte phagocytosis.
Page 6: **Appendix Figure S5.** Analyzing the relationship between the frequency of engulfment events and the developmental fate of surviving oocytes.
Page 7: **Appendix Figure S6.** Labeling germ cell cytoplasm in the *Oct4-CreER*^{T2}*;Rainbow* ovaries.
Page 8: **Appendix Figure S7.** Suppressing the FL formation by Formin inhibitor significantly.
Page 9: **Appendix Figure S8.** Mitochondria numbers were enrichment during oocyte phagocytosis.
Page 10: **Appendix Figure S9.** The expression dynamics of the representative genes in the different cell lineages.
Page 11: **Appendix Figure S10.** Blocking oocyte phagocytosis by 3MA treatment suppressed the growth and the enrichment of mitochondria in the survived oocytes.
Page 12: **Appendix Figure S11.** Deletion *Tex14* in germ cells had no effects on oocyte phagocytosis.

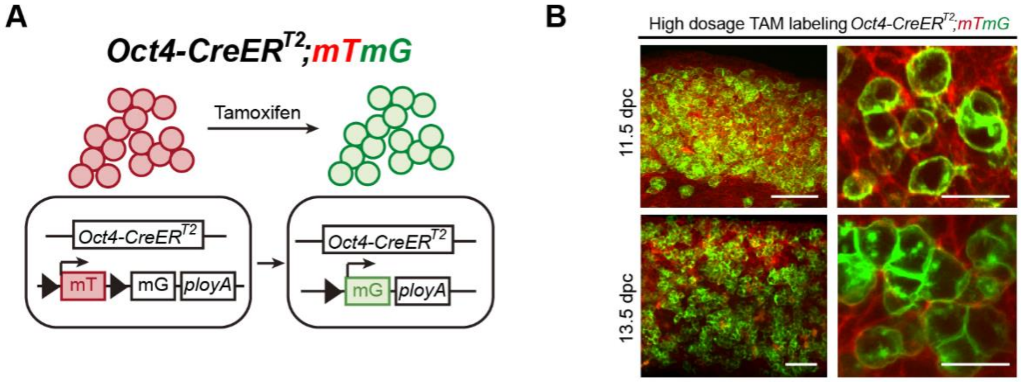

Appendix Figure S1. Labeling germ cell membrane in the *Oct4-CreER^{T2};mTmG* ovaries.

(A) Illustration of tamoxifen (Tam)–induced labeling of germ cells in *Oct4-CreER^{T2};mTmG* ovaries. In *Oct4*-expressing germ cells, the CreER^{T2} recombinase is inactive and the cells express mT, a membrane localized red fluorescent protein. Upon tamoxifen injection, the CreER^{T2} recombinase deletes the mT region and the germ cells switches on the expression of mG, a membrane localized green fluorescent protein. **(B)** Pregnant females carrying *Oct4-CreER^{T2};mTmG* fetus received i.p. injections of tamoxifen at a dose of 50 mg.kg^{-1} BW at 10.5 dpc, and the labeled fetal ovaries were collected at 11.5 dpc and 13.5 dpc, displaying germ cells with GFP-labeled membrane. Scale bar: 50 μm.

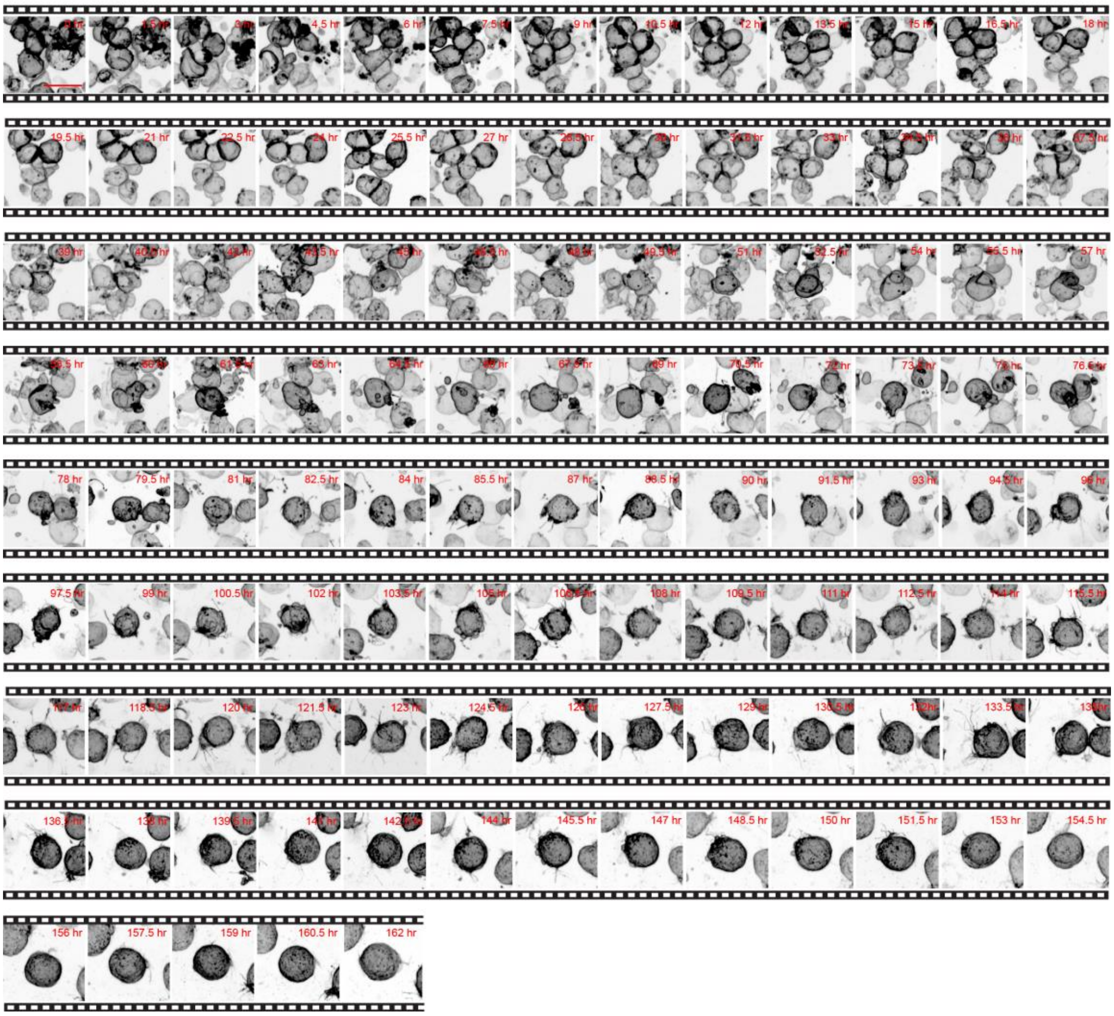

Appendix Figure S2. Time-lapse imaging to trace the development of a group of oocytes.

The developmental dynamics of oocytes were monitored using time-lapse imaging at 1.5-hour intervals over a period of 162 hours. The oocytes were displayed in inverted black/white (b/w) to highlight. Scale bar: 20 μm.

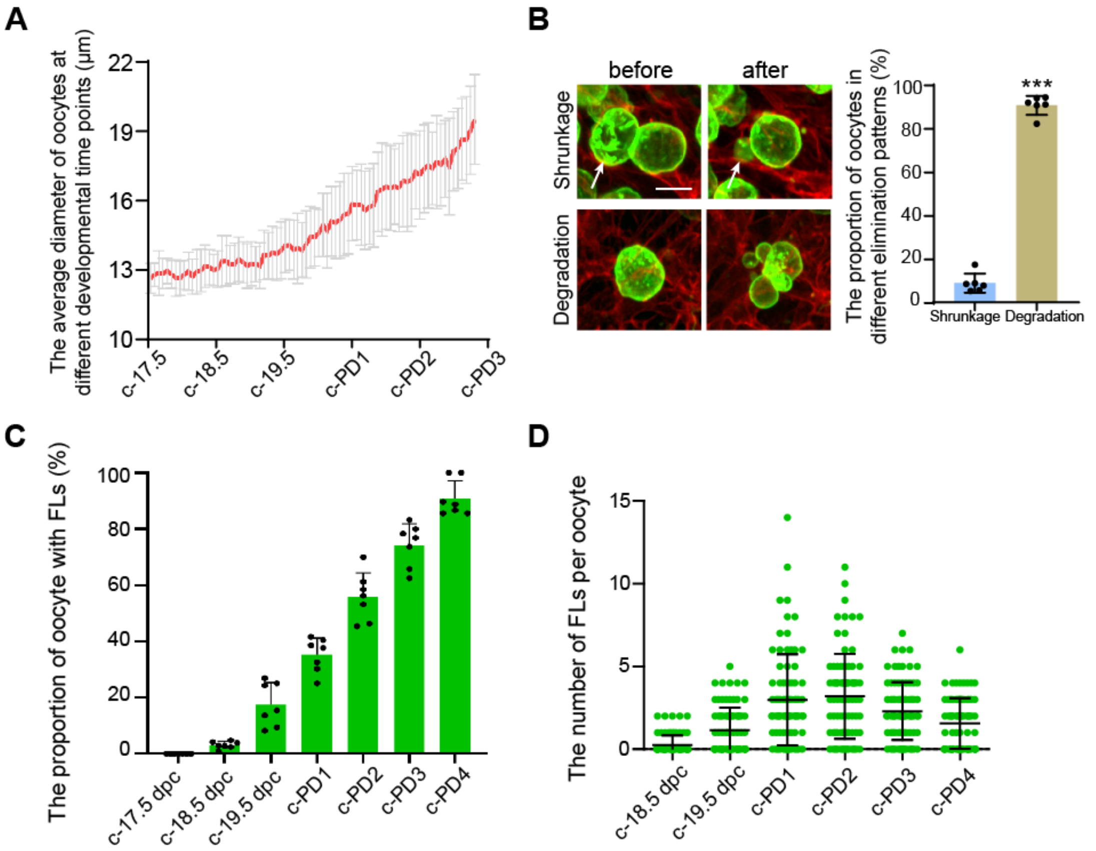

Appendix Figure S3. Quantification of the developmental dynamics and cellular behaviors of oocytes during ovariogenesis.

(A) The average diameter of oocytes was measured during growth from c-17.5 dpc to c- PD3. By randomly tracing 20 surviving oocytes in six ovaries using 4D imaging, the average diameter was recorded every 1.5 hours to generate the growth curve. Results indicated a significant increase in oocyte size predominantly from c-19.5 dpc to c-PD3. **(B)** Quantification of oocyte elimination (seen Figure 2C lane 2 and lane 3) patterns demonstrated that degradation was the primary type of elimination observed. n = 6 ovaries, with more than 15 oocyte eliminations traced per ovary. Scale bar: 10 μm. **(C)** Statistical analysis showed a continuously increasing proportion of surviving oocytes with FL structures from c-18.5 dpc to c-PD4. n = 6 ovaries. **(D)** Counting the number of FLs per oocyte, showing the most active formation of FLs occurred from c-PD1 to c-PD2. n = 80 oocytes at each time point. Data are presented as the mean ± SD. ***P ≤0.001 by two-tailed unpaired Student's t-test.

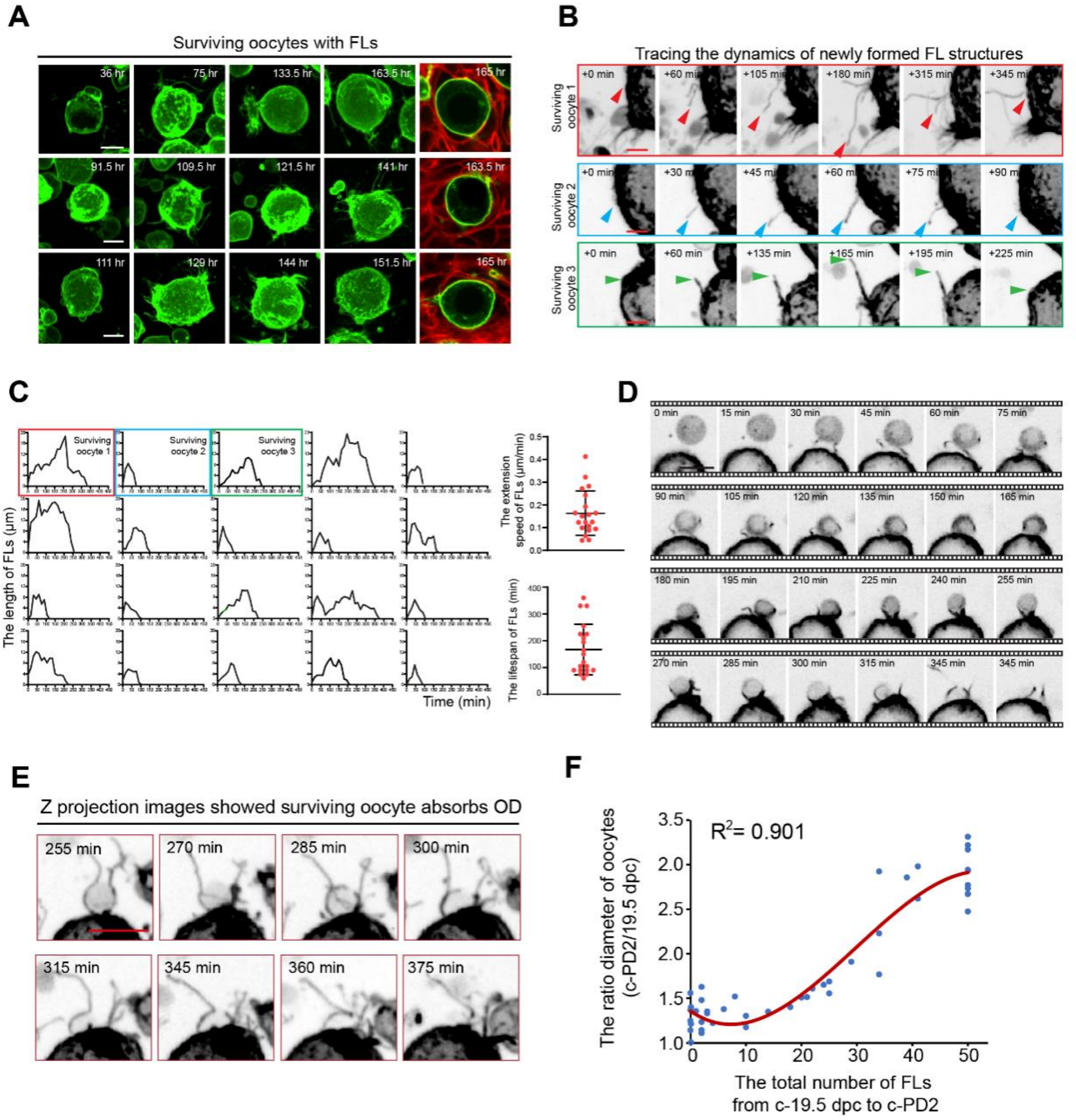

Appendix Figure S4. Recording and analyzing the progress of oocyte phagocytosis.

(A) Tracing the development of surviving oocytes that form ovarian follicles in culture. Showing the formation of FLs on the surviving oocytes at different time points. **(B-C)** Tracing (B) and quantifying (C) the developmental dynamics of newly formed FL structures on surviving oocytes. The time-lapse images showed the dynamics of newly formed FL structures on surviving oocyte at c-PD1 (B). Arrowheads in (B) traced the terminals of FLs. The real-time growth curves of 20 newly formed FLs during 450 min (C, left). Statistical analysis of the extension speed (upper) and the lifespan (bottom) of FLs. n = 20 oocytes (C, right). Data are presented as the mean ± SD. **(D-E)** The time-lapse imaging recorded detailed behaviors of oocytes during oocyte phagocytosis at 15-minute interval. Single Z time-lapse imaging illustrated the detailed process of FL-associated OD engulfment (D), and the Z projection images revealed the surviving oocyte absorbing the ODs with the assistance of FLs (E). Oocytes were inverted to black/white (b/w). **(F)** The statistical curve showed the positive correlation between the growth ratio of surviving oocytes and number of FLs in each oocyte. Scale bar in (A, D, E): 10 μm.; Scale bar in (b): 5 μm.

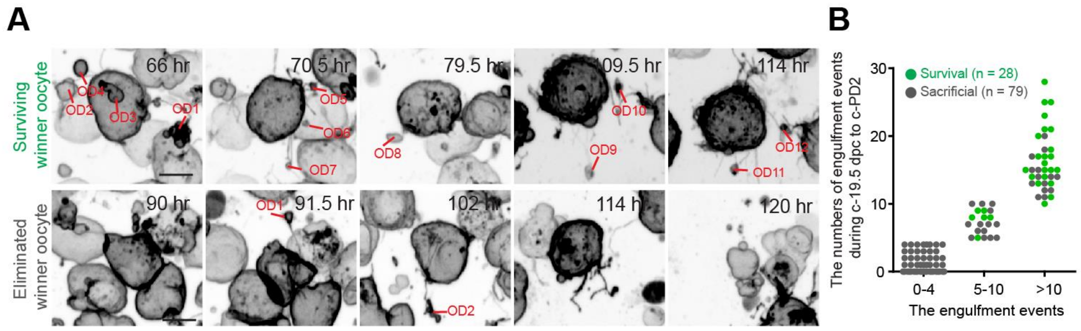

Appendix Figure S5. Analyzing the relationship between the frequency of engulfment events and the developmental fate of surviving oocytes.

(A) Identification of the frequency of potential engulfment events through an in-depth analysis of the 4D time-lapse images (data as shown in Fig 2A-B). Both FL connected ODs and the attached ODs were counted as one engulfment event. Duplicate counts was excluded through continuous observation. A surviving oocyte (upper) and an eliminated oocyte both with FLs (bottom) were chosen to show the criteria for identifying engulfment events during the same 48-hour observation window (from c-19.5 dpc to c-PD2). Scale bar: 10 μm. **(B)** The relationship between the frequency of engulfment events and the fate of oocytes during the 48-hour developmental period. All oocytes that engulfed ≤4 OD fragments uniformly failed to survive at c-PD4. In contrast, surviving oocytes that formed follicles had significantly higher engulfment activity, with an average of 15 OD fragments engulfed during this period. n = 107 oocytes from 6 ovaries.

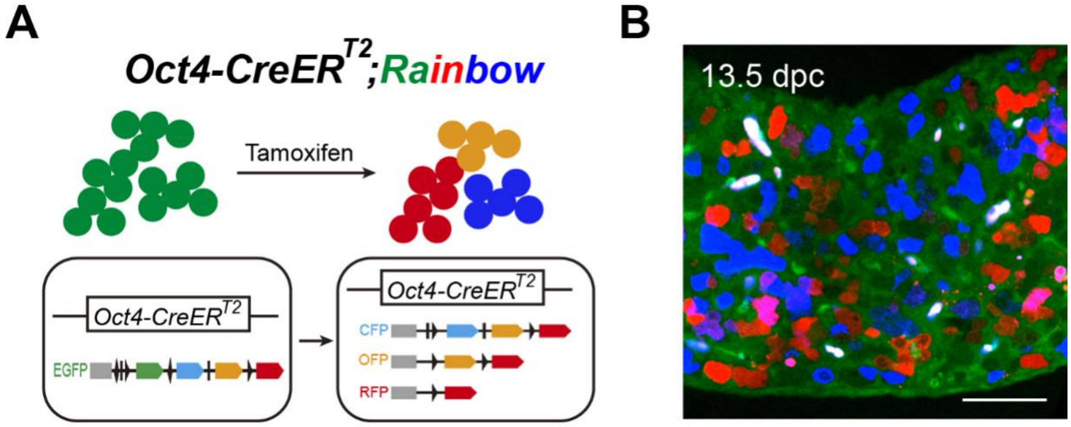

Appendix Figure S6. Labeling germ cell cytoplasm in the *Oct4-CreER^{T2};Rainbow* ovaries.

(A) Illustration of tamoxifen (Tam)–induced labeling of germ cells in *Oct4-CreER^{T2};Rainbow* ovaries. In *Oct4*-expressing germ cells, the CreER^{T2} recombinase is not active and the cells express EGFP. Upon tamoxifen injection, the CreER^{T2} recombinase deletes the EGFP region and switches on a random expression of CFP, OFP or RFP. Thus, the cytoplasm of different *Oct4*-expressing germ cells was labeled with blue, orange or red fluorescence. **(B)** Pregnant females carrying *Oct4-CreER^{T2};Rainbow* fetus received an i.p. injection of tamoxifen at a dose of 50 mg.kg^{-1} BW at 10.5 dpc, and the labeled fetal ovaries were collected at 13.5 dpc, displaying germ cells with random red or blue cytoplasm. Scale bar: 50 μm.

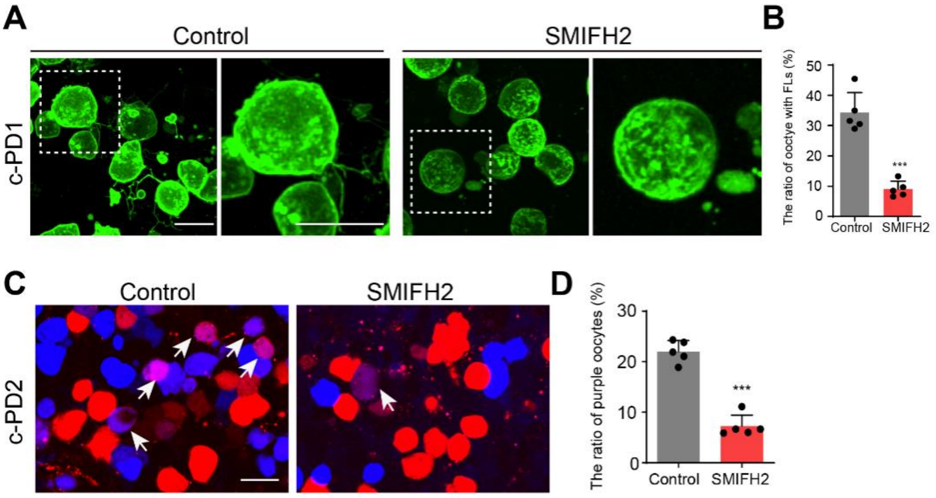

Appendix Figure S7. Suppressing the FL formation by Formin inhibitor significantly decreased the efficiency of cytoplasmic exchange in oocyte phagocytosis.

(A) Illustration of suppression of FL (filopodia-like) formation in surviving oocytes by adding SMIFH2 (20 μM) at c-PD1. **(B)** Statistical analysis showed that a significantly decreased ratio of oocytes with FLs in SMIFH2-treated ovaries compared to the control group at c-PD1. Scale bar: 20 μm. n = 5 ovaries (n > 60 oocytes per ovary). **(C-D)** SMIFH2 treatment resulted in a decreased ratio of oocytes with mixed fluorescent cytoplasm (purple, arrows) at c-PD2. Scale bar: 20 μm. n = 5 ovaries (n > 45 oocytes per ovary). Data are presented as the mean ± SD. ***P <0.001 by two-tailed unpaired Student's t test.

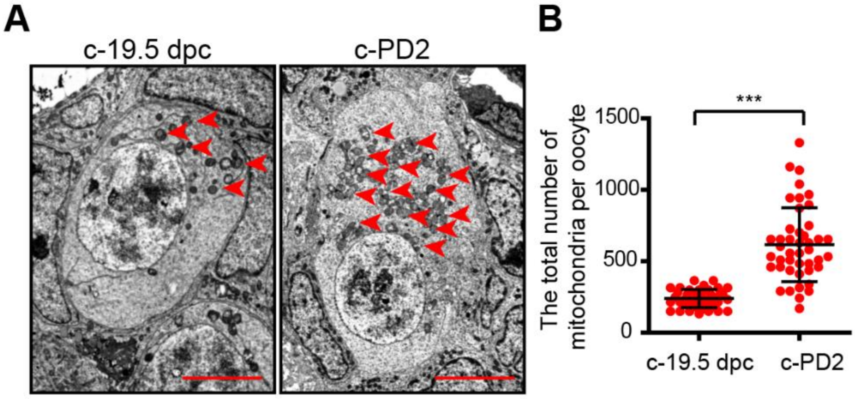

Appendix Figure S8. Mitochondria numbers were enrichment during oocyte phagocytosis. (A) TEM images showing mitochondrial numbers (red arrowheads) in oocytes at c-19.5 and c-PD2. (B) The total number of mitochondria in oocytes at c-PD2 was significantly increased compared to oocytes at c-19.5 dpc. n > 40 oocytes per group. Scale bar: 5 µm. ***P <0.001 by two-tailed unpaired Student's t test.

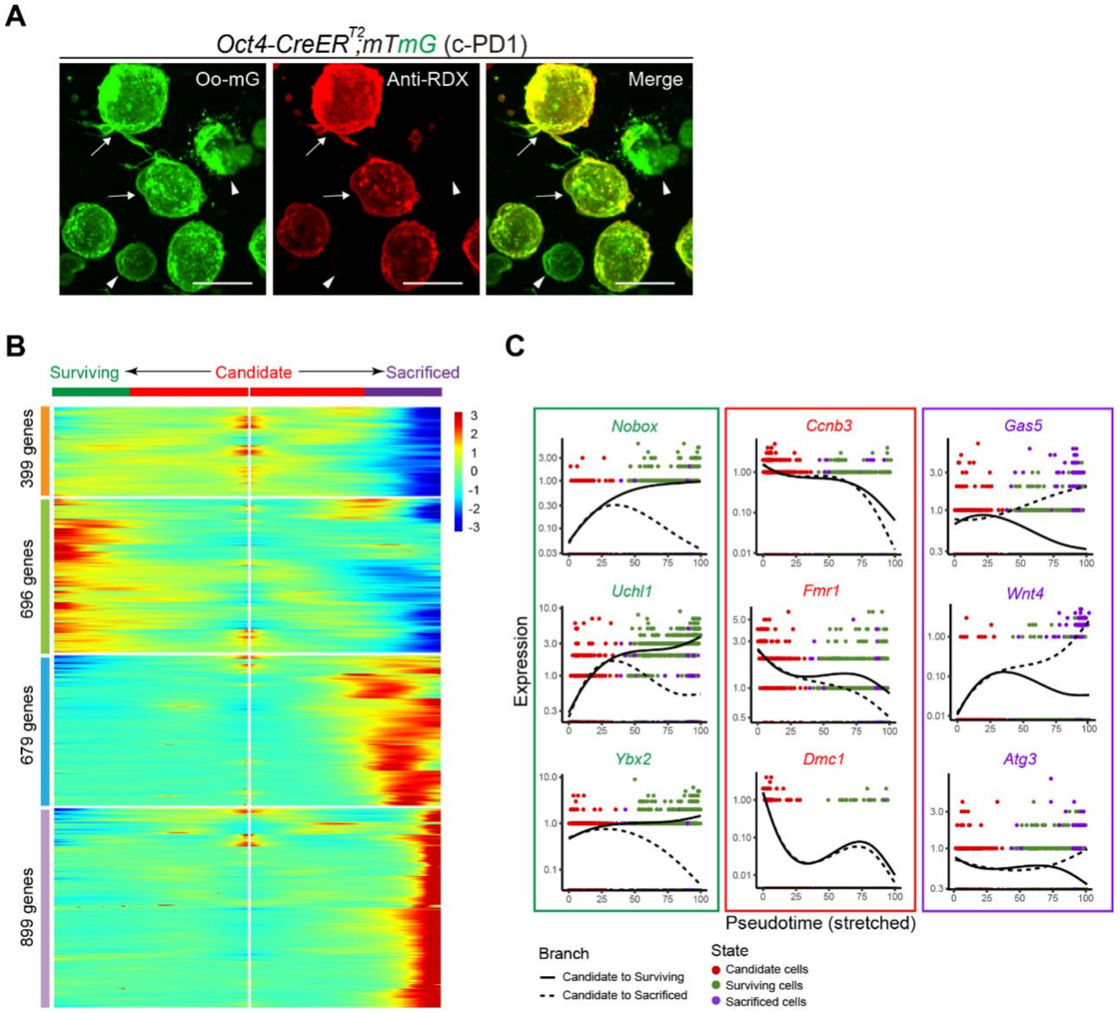

Appendix Figure S9. The expression dynamics of the representative genes in the different cell lineages.

(A) Immunostaining of RDX in developing oocytes at c-PD1. Showing high expression of RDX in the surviving oocytes with FL (arrows) in *Oct4-CreER^{T2};mTmG* ovaries at c-PD1. In contrast, the loser oocytes and ODs (arrowheads) lacked RDX expression. Scale bar: 20 µm. **(B-C)** Heat map (A) and plot (B) of the expression branched dynamics of the representative genes in the different cell lineages in the pseudotime analysis. Expression patterns of the candidate lineage genes *Ccnb3*, *Fmr1*, and *Dmc1*; Surviving lineage expressing follicle related genes *Nobox*, *Uchl1*, and *Ybx2*; and Sacrificed lineage expressing genes *Gas5*, *Wnt4*, and *Atg3*.

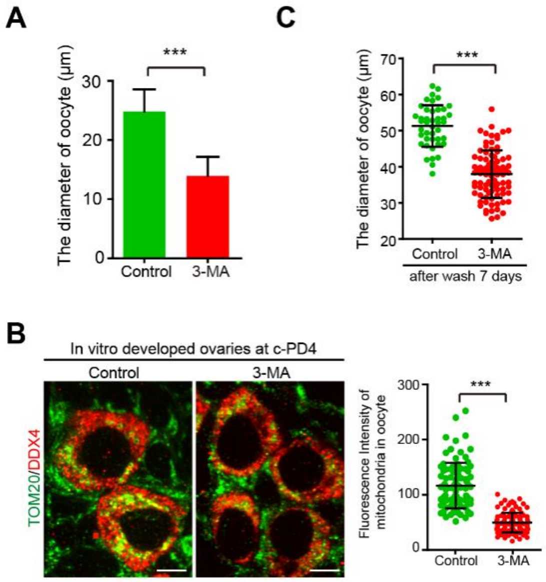

Appendix Figure S10. Blocking oocyte phagocytosis by 3MA treatment suppressed the growth and the enrichment of mitochondria in the survived oocytes.

(A) Quantification of oocyte average diameter exhibited significant growth retardation of oocytes in 3-MA treated ovaries compared to control ovaries at c-PD4. Control: n = 48 oocytes from 4 ovaries, 3-MA: n = 97 oocytes from 4 ovaries. **(B)** 3-MA treatment decreased the density of mitochondria in the surviving oocytes. Red: DDX4, Green: TOM20. Scale bar: 10 μm. Control: n = 101 oocytes from 3 ovaries, 3-MA: n = 106 oocytes from 3 ovaries. **(C)** Quantification of oocyte average diameter demonstrated that oocytes without sacrifice in 3-MA treated ovaries were unable to fully grow. Control: n = 43 oocytes from 3 ovaries, 3-MA: n = 88 oocytes from 3 ovaries. ***P <0.001 by two-tailed unpaired Student's t test.

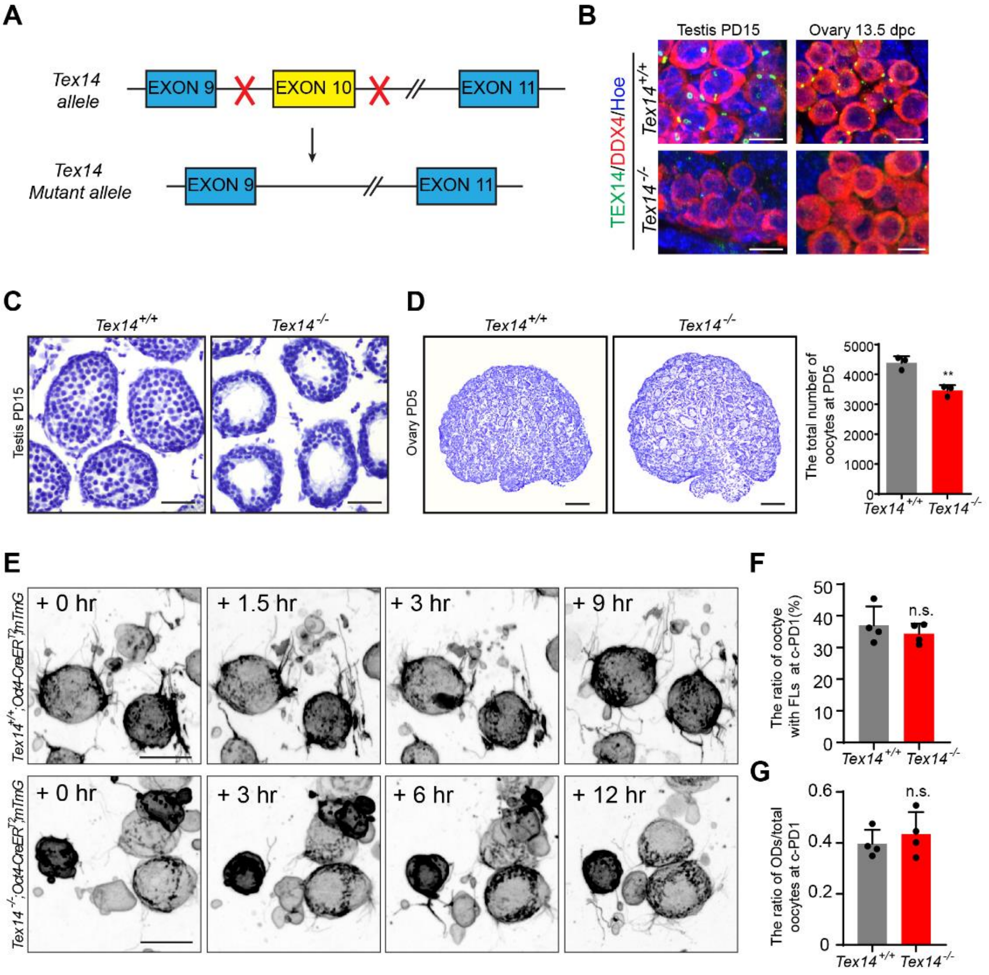

Appendix Figure S11. Deletion *Tex14* in germ cells had no effects on oocyte phagocytosis.
(A) Following the deletion strategy published[63], exon 10 in *Tex14* alleles was targeted for deletion using CRISPR/Cas9. **(B)** Immunofluorescence analysis showed an absence of TEX14-formed intercellular bridges in *Tex14*$^{-/-}$ testes at postnatal day 15 (PD15) and ovaries at 13.5 days post coitum (dpc). TEX14 (green), DDX4 (red). Scale bar: 10 μm. **(C)** Histological analysis revealed abnormal morphology and a decreased number of spermatocytes in *Tex14*$^{-/-}$ male testes at PD15. Scale bar: 50 μm. **(D)** Histological assessment displayed relatively normal ovarian morphology in *Tex14*$^{-/-}$ females at postnatal day 5 (PD5), although follicle numbers were decreased compared to *Tex14*$^{+/+}$ ovaries. Scale bar: 100 μm. n = 3 ovaries. p-value = 0.0046. **(E)** In *Tex14*$^{-/-}$*;Oct4-CreER*^{T2}*;mTmG* ovaries at complementary PD1 (c-PD1), both oocytes with follicle-like structures (FL) and oocyte-derived structures (ODs) were observed, similar to control *Tex14*$^{+/+}$*;Oct4-CreER*^{T2}*;mTmG* ovaries. Scale bar: 20 μm. **(F-G)** Statistical analysis showed no significant difference in the ratio of oocytes with FLs (F) and ODs (G) in *Tex14*$^{-/-}$ ovaries compared to *Tex14*$^{+/+}$ ovaries at c-PD1. p-value (F) = 0.4571; p-value (G) = 0.4942.Data are presented as mean ± SD; n = 4 ovaries (n > 60 oocytes per ovary). n.s. P >0.05, **P <0.01 by two-tailed unpaired Student's t test.
