# Supplementary material for: Cyst-independent oocyte phagocytosis builds the female reproductive reserve in mice
Source: EMBO Rep. 2025 Dec 8;27(1):230–55. doi: 10.1038/s44319-025-00663-7 (PMC12796176; doi:10.1038/s44319-025-00663-7)
Supplement: Supplementary file 7 — Movie EV1 [file 44319_2025_663_MOESM7_ESM.zip › Movie EV1 legend.docx]

**Movie EV1. Developmental dynamics of oocytes in live ovary**

The representative time-lapse movie represents the developmental dynamics of oocytes in live ovaries of *Oct4-CreER^T2^;mTmG* in vitro over a period of 162 hours with images captured at 1.5-hour intervals, from c-17.5 dpc to c-PD4. Scale bars: 50 μm (left and middle views), 10 μm (right view). Green, oocytes; Red, somatic cells. In the middle and right magnified views, GFP on oocytes were inverted to black/white (b/w) for improved clarity.
