# Supplementary material for: Cyst-independent oocyte phagocytosis builds the female reproductive reserve in mice
Source: EMBO Rep. 2025 Dec 8;27(1):230–55. doi: 10.1038/s44319-025-00663-7 (PMC12796176; doi:10.1038/s44319-025-00663-7)
Supplement: Supplementary file 8 — Movie EV2 [file 44319_2025_663_MOESM8_ESM.zip › Movie EV2 legend.docx]

**Movie EV2. Oocytes act as separated single cells in the ovary after c-17.5 dpc**

The time-lapse movie tracks oocyte movement after c-17.5 dpc over a 12-hour period, with images captured at 1.5-hour intervals. It shows connected oocytes moving in different directions, confirming that oocytes behave as individual single cells rather than as connected cysts. Scale bar: 10 μm. Green, oocytes; Red, somatic cells.
