# Supplementary material for: Cyst-independent oocyte phagocytosis builds the female reproductive reserve in mice
Source: EMBO Rep. 2025 Dec 8;27(1):230–55. doi: 10.1038/s44319-025-00663-7 (PMC12796176; doi:10.1038/s44319-025-00663-7)
Supplement: Supplementary file 9 — Movie EV3 [file 44319_2025_663_MOESM9_ESM.zip › Movie EV3 legend.docx]

**Movie EV3. Formation dynamics of ODs from sacrificed oocytes**

The movie depicts the dynamics of OD formation from a sacrificed oocyte (marked with an asterisk), which breaks into small vesicles with intact membranes. Starting point: c-19.5 dpc. Scale bar: 10 μm. All oocytes were inverted to black/white (b/w).
