# Supplementary material for: Cyst-independent oocyte phagocytosis builds the female reproductive reserve in mice
Source: EMBO Rep. 2025 Dec 8;27(1):230–55. doi: 10.1038/s44319-025-00663-7 (PMC12796176; doi:10.1038/s44319-025-00663-7)
Supplement: Supplementary file 11 — Movie EV5 [file 44319_2025_663_MOESM11_ESM.zip › Movie EV5 legend.docx]

**Movie EV5. Detailed behaviors of surviving oocytes in live imaging 2**

The time-lapse movie captures the detailed behaviors of oocytes during oocyte phagocytosis, with images recorded at 15-minute interval. It shows the surviving oocyte 2 absorbing the ODs (arrows) with the assistance of FLs. Starting point: c-PD1. Scale bar: 10 μm. GFP on oocytes were inverted to black/white (b/w) for improved clarity.
