# Supplementary material for: Cyst-independent oocyte phagocytosis builds the female reproductive reserve in mice
Source: EMBO Rep. 2025 Dec 8;27(1):230–55. doi: 10.1038/s44319-025-00663-7 (PMC12796176; doi:10.1038/s44319-025-00663-7)
Supplement: Supplementary file 13 — Movie EV7 [file 44319_2025_663_MOESM13_ESM.zip › Movie EV7 legend.docx]

**Movie EV7. The absorbed ODs behaviors of surviving oocytes** **in fresh ovary at PD1**

The time-lapse movie captures the detailed behaviors of oocytes during oocyte phagocytosis in fresh *Oct4-CreER^T2^;mTmG* ovary at PD1, with images recorded at 15-minute interval. It shows the surviving oocyte absorbing the ODs (arrows) with the assistance of FLs. Starting point: PD1. Scale bar: 10 μm.
