# Supplementary material for: Cyst-independent oocyte phagocytosis builds the female reproductive reserve in mice
Source: EMBO Rep. 2025 Dec 8;27(1):230–55. doi: 10.1038/s44319-025-00663-7 (PMC12796176; doi:10.1038/s44319-025-00663-7)
Supplement: Supplementary file 14 — Movie EV8 [file 44319_2025_663_MOESM14_ESM.zip › Movie EV8 legend.docx]

**Movie EV8. No cytoplasm exchange occurs through oocyte attachment**

The attachment of oocytes with different fluorescence is traced in the live ovaries of *Oct4-CreER^T2^;Rainbow* female, showing that no cytoplasm mixing occurs following attachment (arrowheads) between oocytes. Starting point: c-18.5 dpc. Scale bar: 5 μm.
