# Supplementary material for: Cyst-independent oocyte phagocytosis builds the female reproductive reserve in mice
Source: EMBO Rep. 2025 Dec 8;27(1):230–55. doi: 10.1038/s44319-025-00663-7 (PMC12796176; doi:10.1038/s44319-025-00663-7)
Supplement: Supplementary file 15 — Movie EV9 [file 44319_2025_663_MOESM15_ESM.zip › Movie EV9 legend.docx]

**Movie EV9. Cytoplasmic exchange during oocyte phagocytosis in a live ovary**

The time-lapse movie captures a blue surviving oocyte (arrow) absorbing red ODs (arrowheads), resulting in a gradual cytoplasmic color change from blue to purple. Starting point: c-PD1. Scale bar: 10 μm.
