# Supplementary material for: Cyst-independent oocyte phagocytosis builds the female reproductive reserve in mice
Source: EMBO Rep. 2025 Dec 8;27(1):230–55. doi: 10.1038/s44319-025-00663-7 (PMC12796176; doi:10.1038/s44319-025-00663-7)
Supplement: Supplementary file 20 — Expanded View Figures [file 44319_2025_663_MOESM20_ESM.pdf]

Expanded View Figures

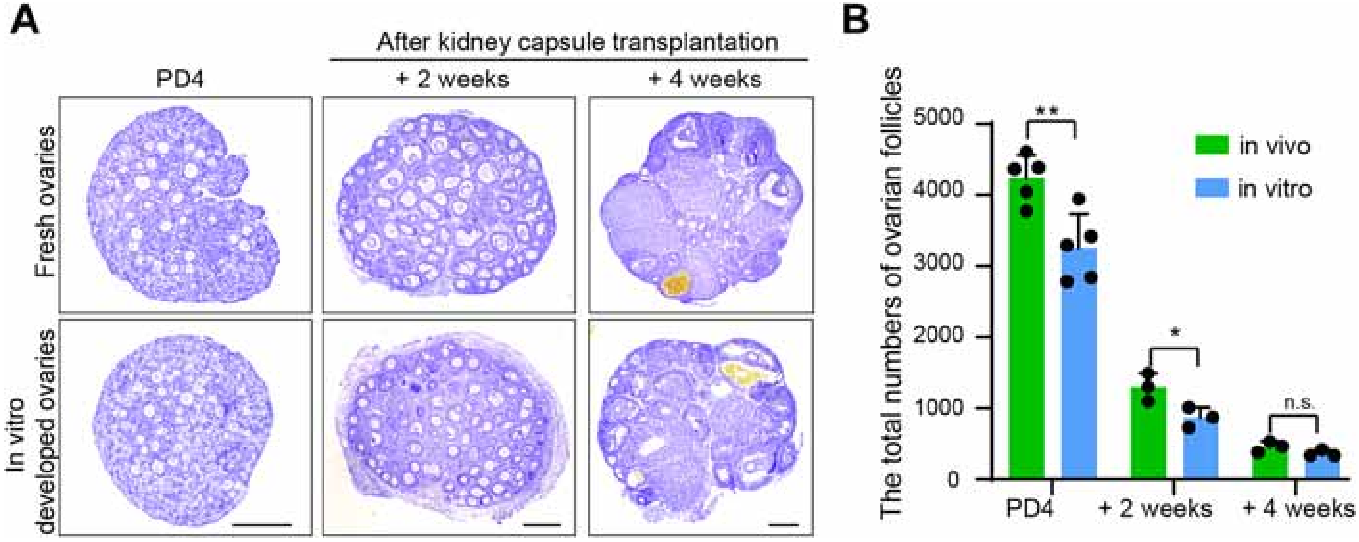

Figure EV1. Normal follicle development in cultured ovaries after allo-transplantation.

(**A**) Histological analysis displaying a regular distribution of follicles in both fresh and cultured ovaries throughout different developmental stages after kidney capsule transplantation. Scale bar: 100 µm. (**B**) Follicle counting detection revealed a slight reduction in the number of follicles in cultured ovaries compared to those in fresh ovaries at c-PD4 and 2 weeks post-transplantation. $n \geq 3$ ovaries at every time point. The data were presented as the mean ± SD. Statistical significance is determined using a two-tailed unpaired Student's *t*-test; PD4: *p* value = 0.0065; 2 weeks: *p* value = 0.013; 4 weeks: *p* value = 0.148. n.s. $P > 0.05$, $^{*}P \leq 0.05$, $^{**}P \leq 0.01$.

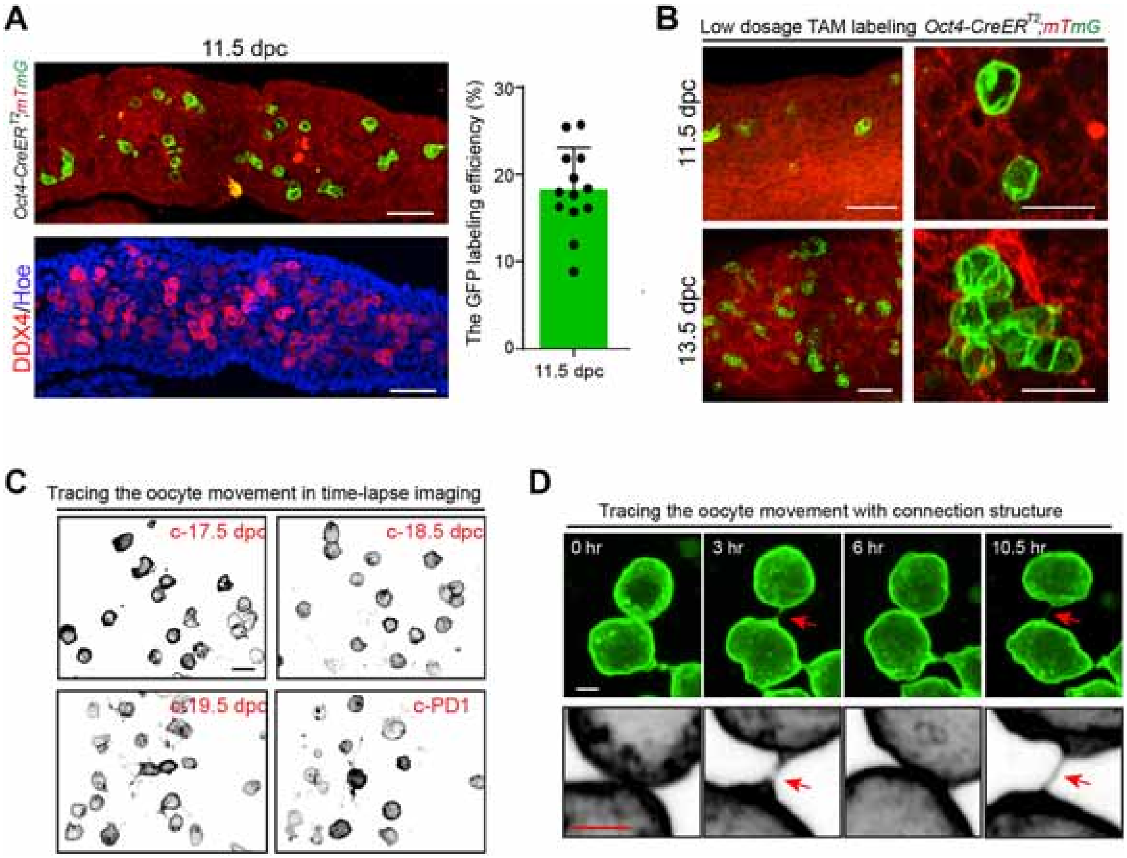

Figure EV2. Tracing the oocyte movement and separation in the *Oct4-CreER^{T2};mTmG* ovaries after low dosage Tam treatment.

(**A**) Evaluation of the labeling efficiency in oocytes within *Oct4-CreER^{T2};mTmG* ovaries at 11.5 dpc. Pregnant females carrying *Oct4-CreER^{T2};mTmG* fetus were treated with tamoxifen (20 mg·kg^{-1} BW) at 10.5 dpc (left upper). Immunofluorescent staining of oocytes using DDX4 antibody in the same sections (left bottom). Statistical analysis of oocyte labeling efficiency, determined by the ratio of GFP-positive oocytes to the total number of DDX4-positive cells. Data collected from 13 sections across four ovaries are presented as mean ± SD (right). (**B**) Showing the labeling efficiency of oocytes in the *Oct4-CreER^{T2};mTmG* ovaries. Showing the labeled single germ cells at 11.5 dpc and separated cysts at 13.5 dpc in the ovaries after a low dosage of Tam treatment. Scale bar: 50 µm. (**C**) Tracing the movement of labeled oocytes demonstrated majority of oocytes moving as single cells from c-17.5 dpc to c-PD1. Oocytes were displayed in inverted black/white (b/w) to highlight. Scale bar: 20 µm. (**D**) Showing the criteria for identifying single oocytes. Oocytes with any potential connections (arrows) were excluded from the counting of single cells. Scale bar: 5 µm.

 © The Author(s)

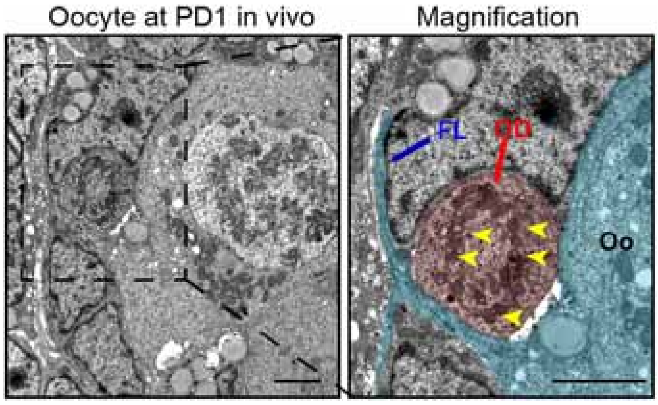

Figure EV3. Oocyte phagocytosis is observed in a TEM image at PD1.

Representative TEM image of an oocyte at PD1 in the ovary, showing an oocyte (cyan) containing FL structures (blue) and mitochondria (yellow arrowheads) within an OD (red). Scale bar: 2 μm.

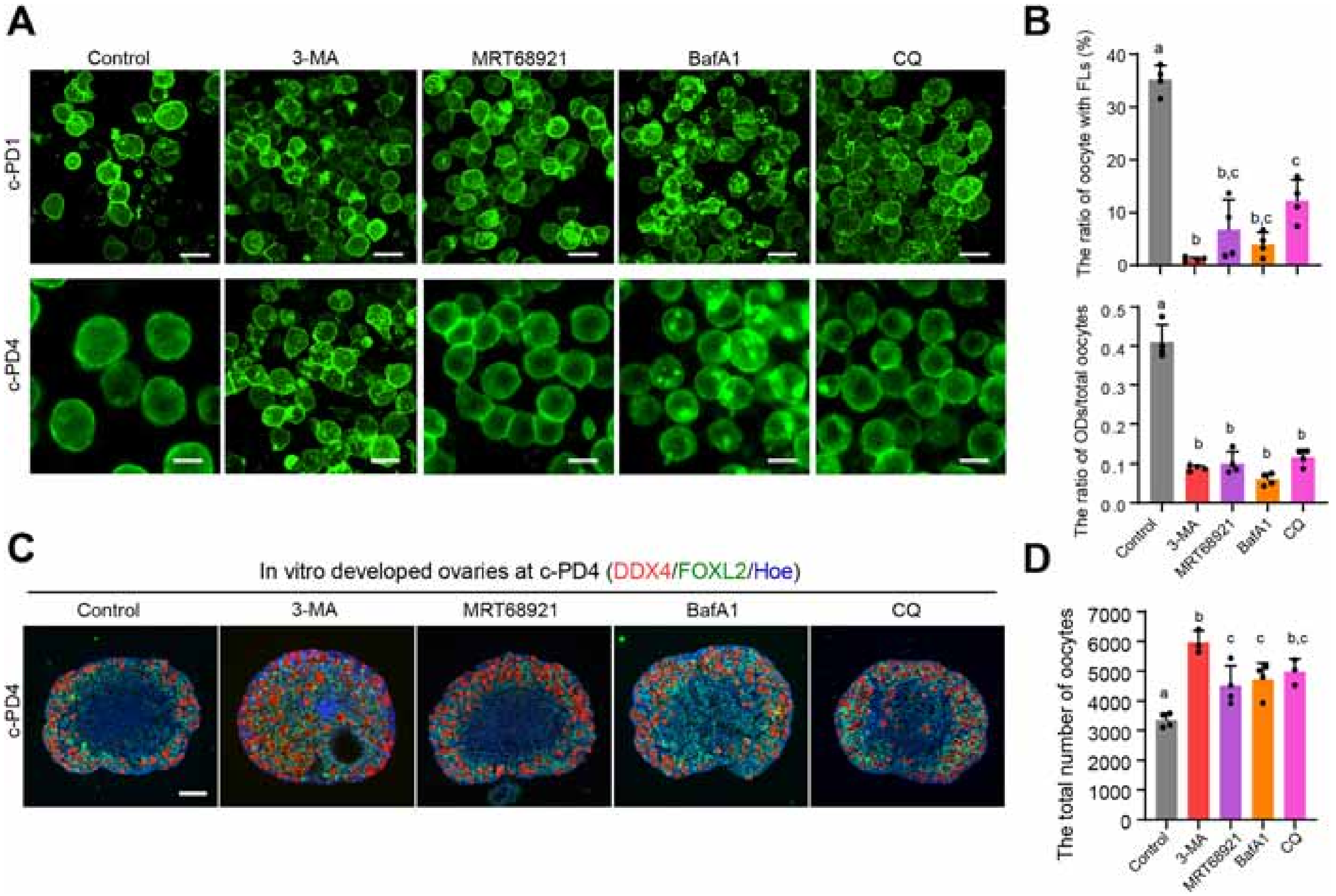

Figure EV4. Impact of autophagy inhibition on oocyte phagocytosis during ovariogenesis.

(**A**) The analysis of oocyte phagocytosis and development in cultured ovaries treated from c-17.5 to c-PD4 with autophagy inhibitors (3MA, MRT68921, BafA1, and CQ). Autophagy inhibition consistently reduced sacrifice and phagocytosis at c-PD1 and increased the survival of oocytes by c-PD4. Scale bar: 20 μm. (**B**) Statistical evaluations showed that suppression of autophagy significantly diminishes oocyte sacrifice and phagocytosis at c-PD1, demonstrating a sharp decline in the ratio of surviving oocytes with FLs and a decrease in the formation of ODs from sacrificed oocytes. The analysis included four ovaries per group, evaluating more than 70 oocytes per ovary. FL: control vs. 3-MA: *p* value = 5.9E-09; control vs. MRT: *p* value = 6.7E-08; control vs. BafA1: *p* value = 1.9E-08; control vs. CQ: *p* value = 1.1E-06; 3-MA vs. MRT: *p* value = 0.21; 3-MA vs. BafA1: *p* value = 0.79; 3-MA vs. CQ: *p* value = 0.003; MRT vs. BafA1: *p* value = 0.80; MRT vs. CQ: *p* value = 0.22; BafA1 vs. CQ: *p* value = 0.05. OD: control vs. 3-MA: *p* value = 2.3E-10; control vs. MRT: *p* value = 4.1E-10; control vs. BafA1: *p* value = 7.2E-09; control vs. CQ: *p* value = 7.8E-08; 3-MA vs. MRT: *p* value = 0.96; 3-MA vs. BafA1: *p* value = 0.54; 3-MA vs. CQ: *p* value = 0.66; MRT vs. BafA1: *p* value = 0.23; MRT vs. CQ: *p* value = 0.94; BafA1 vs. CQ: *p* value = 0.07. (**C**, **D**) Immunofluorescent staining of the ovarian morphology (**C**) and oocyte number counting (**D**) at c-PD4 showed an increased number of surviving oocytes in ovaries treated with autophagy inhibitors. Red - DDX4; Green - FOXL2; Blue - Hoechst. Scale bar: 100 μm. Data were from at least three ovaries per group. Data were presented as the mean ± SD. control vs. 3-MA: *p* value = 7.25E-05; control vs. MRT: *p* value = 0.034; control vs. BafA1: *p* value = 0.011; control vs. CQ: *p* value = 0.0054; 3-MA vs. MRT: *p* value = 0.012; 3-MA vs. BafA1: *p* value = 0.035; 3-MA vs. CQ: *p* value = 0.155; MRT vs. BafA1: *p* value = 0.96; MRT vs. CQ: *p* value = 0.71; BafA1 vs. CQ: *p* value = 0.95. Statistical significance was determined by ANOVA tests. P (a, b) < 0.05, P (a, c) < 0.05, P (b, c) < 0.05.

 © The Author(s)
